# Lipoxin A4 attenuates MSU-crystal-induced NLRP3 inflammasome activation through suppressing Nrf2 thereby increasing TXNRD2

**DOI:** 10.3389/fimmu.2022.1060441

**Published:** 2022-12-08

**Authors:** You Zhou, Yongjun Chen, Xiaowu Zhong, Hongtao Xia, Mingcai Zhao, Mengyuan Zhao, Lei Xu, Xiaolan Guo, Chong-Ge You

**Affiliations:** ^1^ Laboratory Medicine Center, Lanzhou University Second Hospital, Lanzhou, China; ^2^ Department of Medical Laboratory, Central Hospital of Suining, Suining, China; ^3^ Translational Medicine Research Center, North Sichuan Medical College, Nanchong, China

**Keywords:** lipoxinA4, MSU, gout, NLRP3 inflammasome, ROS, Nrf2, TXNRD2

## Abstract

Gout is a common inflammatory disease. The activation of NLRP3 inflammasome induced by monosodium urate (MSU) crystals has a critical role in gout, and its prevention is beneficial for patients. Lipoxin A4 (LXA4) is an endogenous lipoxygenase-derived eicosanoid mediator with powerful anti-inflammatory properties. However, whether LXA4 can suppress NLRP3 inflammasome activation induced by MSU crystals remains unclear. This study aimed to investigate the protective effect of LXA4 on MSU-crystal-induced NLRP3 inflammasome activation and its underlying molecular mechanisms. We found that LXA4 inhibited MSU-crystal-induced NLRP3 inflammasome activation, interleukin (IL)-1β maturation, and pyroptosis. More specifically, LXA4 suppressed the assembly of the NLRP3 inflammasome, including oligomerization and speck formation of ASC, and ASC-NLRP3 interaction. Furthermore, LXA4 suppressed oxidative stress, the upstream events for NLRP3 inflammasome activation, as evidenced by the fact that LXA4 eliminated total reactive oxygen species (ROS) generation and alleviated nicotinamide adenine dinucleotide phosphate (NADPH) oxidase activation and mitochondrial dysfunction. However, LXA4 also depressed the Nrf2 activation, a critical molecule in the antioxidant pathway, and then exerted an inhibitory impact on Klf9 expression and promotional impact on TXNRD2 expression, two molecules located downstream of Nrf2 in sequence. Knockdown of TXNRD2 reversed the LXA4-induced depression of ROS and NLRP3 inflammasome. Moreover, LXA4 alleviated joint inflammation and decreased the production of cleaved caspase-1 and matured IL-1β in gouty arthritis rats. Taken together, our findings demonstrate that LXA4 can attenuate MSU-crystal-induced NLRP3 inflammasome activation, probably through suppressing Nrf2 activation to increase TXNRD2 expression. The present study highlights the potential of LXA4 as an attractive new gout treatment candidate.

## Introduction

Gout is an inflammatory disease characterized by acute and severe inflammation caused by monosodium urate (MSU) crystals deposition in the articular and periarticular tissues (1). The morbidity of gout has been gradually increasing on a yearly basis (2, 3). Nonsteroidal anti-inflammatory drugs (NSAIDs), colchicine, and corticosteroids are the most common drugs used to treat gout flares and prevent future attacks; yet, these medications have also been associated with certain complications. The pathological mechanism leading to gout is still not fully understood. Previous studies confirmed that the deficiency of endogenous anti-inflammatory mediators might be involved in its pathogenesis (4, 5). Therefore, taking appropriate endogenous anti-inflammatory mediators as therapeutic agents may benefit patients with gout owing to targeted regulation of inflammatory balance and avoidance of drug side effects.

NLR family pyrin domain-containing 3 (NLRP3) inflammasome is essential in immune and inflammatory responses (6). Upon activation, the activated NLRP3 inflammasome serves as a platform for the maturation and release of pro-inflammatory mediators, subsequently leading to a series of physiological effects (7). Incorrect activation of NLRP3 inflammasome has been detected in multiple human diseases, such as type 2 diabetes, neuroinflammation, and gout (8–11). Previous studies have suggested that the activation of NLRP3 inflammasome induced by MSU crystal can result in pyroptosis and secretion of interleukin (IL)-1β, exacerbating inflammatory damage and progression of gout (10, 11). The complete activation of NLRP3 inflammasome relies on the priming signal (the first signal) and the activation signal (the second signal) (7, 12). As a common activator of the second signal (11), MSU crystals can promote the assembly of NLRP3 inflammasome involved in the binding of NLRP3 with apoptosis-associated speck-like protein containing a CARD domain (ASC) and recruitment of pro-caspase-1 (12). There is accumulating evidence demonstrating that taking NLRP3 inflammasome as a target for intervention is a feasible strategy to attenuate MSU crystal-triggered inflammation (7, 10, 11).

Oxidative stress, which results from an imbalance between antioxidant defense mechanisms and reactive oxygen species (ROS) production, is a critical signaling intermediate that drives NLRP3 inflammasome activation (12, 13); this signaling mechanism has been proven in inflammatory responses induced by MSU crystal (14–16). Upon abnormal ROS accumulation induced by MSU crystal, NLRP3 inflammasome assembly can be evoked, followed by activation of caspase-1 and the subsequent release of IL-1β (12). The ROS inhibition can bring a protective effect on the inflammation triggered by MSU crystal. Nuclear factor E2-related factor 2 (Nrf2) is a classical and pivotal master regulator of protective antioxidant and anti-inflammatory responses (17–21). As an inducible transcription factor, activated Nrf2 can exert an antioxidant role by activating and encoding antioxidant proteins, which may suppress NLRP3 inflammasome activation (17, 18). The inhibition of Nrf2 induced by a noxious stimulus is a common pathological cause for some inflammatory diseases (17). However, although MSU crystal can trigger ROS generation, evoking IL-1β/NLRP3 inflammasome depends on Nrf2 activation (11, 20). The intervention of MSU crystal-triggered NLRP3 inflammasome activation may involve a recondite mechanism.

With MSU crystals triggering an inflammatory response, secretion of inflammatory cytokines and arachidonic acid and prostaglandin, protective materials for inflammation are increased (22, 23). Lipoxin A4 (LXA4) is a metabolite for arachidonic acid that naturally exists in our body (23, 24). LXA4 can fight inflammatory response and is a “brake signal” of inflammation (23). Nevertheless, LXA4 exerts its anti-inflammatory role by relying on inhibiting oxidative stress in general (18, 19). Different studies have shown that LXA4 attenuates inflammatory damage by suppressing ROS generation (17–19). Previous studies have shown that LXA4 exhibits a favorable role in oxidative stress and subsequent inflammation by activating Nrf2 in acute lung injury, ischemia–reperfusion injury, and spinal cord injury (17–19). However, the effect of LXA4 on the ROS/NLRP3 inflammasome induced by MSU crystal has not been investigated.

Given that LXA4 grasps anti-inflammatory and antioxidant activities in numerous disease models, we hypothesized that LXA4 could alleviate MSU-crystal-induced NLRP3 inflammasome activation. In this study, we evaluated the inhibitory role of LXA4 on MSU crystals triggered NLRP3 inflammasome activation *in vivo* and *in vitro* and explored the possible mechanism of action.

## Material and methods

### Subjects

A total of 66 male gout patients from Lanzhou University Second Hospital from January 2020 to December 2020 were enrolled in this study. Patients had no acute inflammatory diseases except gouty arthritis and no history of infection, cancer, or other autoimmune diseases. Patients with acute gout (AG) and intercritical gout (IG) were selected based on the American College of Rheumatology (ACR) classification criteria; IG patients were those with complete remission of acute gout with normal erythrocyte sedimentation rate or C-reactive protein. Moreover, age-matched male individuals admitted for regular physical examinations at Lanzhou University Second Hospital were selected as healthy controls (HCs).

This study was approved by the Ethics Committee of the Lanzhou University Second Hospital, and all patients provided informed consent.

### Cell culture and stimulation

Human monocytic leukemia (THP-1) cells were grown in Roswell Park Memorial Institute (RPMI)-1640 culture medium supplemented with 10% fetal bovine serum (FBS) and 1% penicillin/streptomycin (PYG0016; Boster Bio, Wuhan, China). Cells were cultured in a humidified atmosphere containing 5% CO_2_/95% air at 37°C. THP-1 cells were treated with 100 ng/ml phorbol myristate acetate (PMA; S1819, Beyotime Biotechnology, Shanghai, China) for 24 h to differentiate into macrophages.

Bone-marrow-derived macrophages (BMDMs) were obtained *via* the differentiation of bone marrow progenitors from the tibia and femur of C57BL/6J mice in Dulbecco’s modified Eagle’s medium (DMEM) with L929 supernatant (20%), 10% FBS, and 1% penicillin/streptomycin for 7 days. The culture medium was refreshed every other day. The BMDM purity was assessed by flow cytometry using CD11b and F4/80 antibodies.

BMDMs and THP-1 macrophages were primed with 300 ng/ml lipopolysaccharide (LPS) (S1732; Beyotime Biotechnology, Shanghai, China) for 3 h and then pretreated with different concentrations of LXA4 (GC18552; GlpBio, Montclair, CA, USA) for 1 h, and subsequently, 100 μg/ml MSU crystal was added to stimulate for 24 h. MSU crystals were prepared as previously described (10).

For inhibitor experiments, THP-1 macrophages were primed with 300 ng/ml LPS for 3 h and then pretreated with NAC (S0077; Beyotime Biotechnology, Shanghai, China), MitoTEMPO (HY-112879; MCE, NJ, USA), and DPI (HY-100965; MCE, NJ, USA) for 1 h, followed by 100 μg/ml MSU crystal stimulation for 24 h. Moreover, THP-1 macrophages primed with LPS were pretreated with 150 nM LXA4 and tBHQ (HY-100489; MCE, NJ, USA) or auranofin (HY-B1123; MCE, NJ, USA) for 1 h, followed by 100 μg/ml MSU stimulation for 24 h.

### Cytokine measurements

Cytokine levels in serum or conditioned media were detected using an ELISA kit following the manufacturer’s instructions, including LXA4, tumor necrosis factor alpha (TNF-α) (EMC102a; NeoBioScience, Shenzhen, China) and IL-1β (EMC001b; NeoBioScience, Shenzhen, China).

### Western blotting

Proteins for Western blotting were prepared as previously described (25) or performed according to the manufacturer’s instructions from Membrane and Cytosol Protein Extraction Kit (P0033; Beyotime Biotechnology, Shanghai, China) and Nuclear and Cytoplasmic Protein Extraction Kit (P0028; Beyotime Biotechnology, Shanghai, China). Proteins were first separated, transferred, and blocked, after which membranes were incubated with antibodies (see **Supplementary Materials**).

### ASC oligomerization and ASC-speck formation

BMDMs or THP-1 macrophages were treated as described above. Total cells were lysed in cold radioimmunoprecipitation assay (RIPA) buffer (P0013B; Beyotime Biotechnology, Shanghai, China) containing 0.5%Triton X-100, 50 mM Tris–HCl, and 1 mM dithiothreitol for 30 min. The lysates were centrifuged at 330*×g* for 10 min at 4°C, and then, the pellets were collected and washed twice with ice-cold phosphate buffered saline (PBS). The pellets were then resuspended in 500 μl cold PBS containing 4 mM disuccinimidyl suberate (DSS) (S1885; Sigma, St. Louis, MO, USA) and incubated with rotation at room temperature for 30 min. The samples were centrifuged at 1,000*×g* for 10 min to obtain cross-linked pellets, after which Western blotting was performed to analyze ASC oligomerization.

THP-1 macrophages were fixed in 4% paraformaldehyde and then permeabilized with ice-cold methanol. Samples were then incubated with an anti-ASC antibody (1:100) overnight at 4°C and Alexa Fluor 488 goat-anti-mouse IgG (1:100) for 1 h at room temperature. Finally, samples were then stained with 4′,6-diamidino-2-phenylindole (DAPI) (D8200; Solarbio Science, Beijing, China). ASC-speck formation was imaged using confocal laser scanning microscopy (Olympus, Tokyo, Japan).

### Co-immunoprecipitation

BMDMs and THP-1 macrophages treated as described above were lysed, and the lysis was collected. The samples were incubated with the antibody against ASC (1:100) and Nek7 (1:100) overnight at 4°C to obtain antibody–target protein complexes, and the complexes were gathered with Protein G agarose beads for 2 h at 4°C. Next, sediment was resuspended in 1× sodium dodecyl sulfate (SDS) loading buffer and analyzed by Western blotting.

### Pyroptosis cell imaging

THP-1 macrophages were seeded in 24-well plates and treated as described above. Cells were stained with propidium iodide (8 μg/ml) and Hoechst dye (8 μg/ml) (C0003; Beyotime Biotechnology, Shanghai, China) and imaged using a fluorescent microscope (Olympus, Tokyo, Japan). PI-positive cells suggested cell death.

### LDH release assay

THP-1 macrophages were seeded in 12-well plates and treated as described above. Then, 120 μl conditioned supernatant was transferred to 96-well plates and mixed with 60 μl LDH Cytotoxicity Assay Kit reagent (C0016; Beyotime Biotechnology, Shanghai, China) and incubated for 30 min at 37°C. The absorbance was determined at 490 nm using a microplate reader. The cellular LDH release was calculated following the manufacturer’s instructions.

### Intracellular chloride and potassium ion measure

Intracellular chloride ions were detected by MQAE (S1082; Beyotime Biotechnology, Shanghai, China), a fluorescence probe for chloride ions. A higher fluorescence intensity indicated a lower level of chloride ions and *vice versa*. Intracellular potassium ions were detected by EPG-2 AM (MX4513; Maokang Biotechnology, Shanghai, China), a fluorescence indicator for potassium ions.

THP-1 macrophages treated as described above were stained following manufacturer’s instructions. The fluorescence images of the cell-loaded probe were captured using confocal laser scanning microscopy, and the Image software was applied to evaluate fluorescence intensity.

### Cellular ROS measure

THP-1 macrophages were seeded in 12-well plates and treated as described above. Next, cells were incubated with 10 μM 2,7-dichlorodihydrofluorescein diacetate (DCFH-DA) (S0033S; Beyotime Biotechnology, Shanghai, China), a fluorescent probe, for 30 min at 37°C. Fluorescence images of cell-loaded probe were captured using a fluorescent microscope, and fluorescence intensity was measured by a microplate reader.

For the flow cytometry assay, THP-1 macrophages were treated and loaded with DCFH-DA. The cells were collected and suspended in ice-cold PBS, and the flow cytometry and FlowJo software were performed to analyze the level of ROS.

### Measurement of NADPH oxidase activity

Nicotinamide adenine dinucleotide phosphate (NADPH) oxidase activity was determined using lucigenin-enhanced chemiluminescence as previously described (26). THP-1 macrophages were lysed. Samples (30 μl) were then incubated with 300 μl reaction buffer containing 5 mM K_2_HP0_4_, 1 mM EGTA, 150 mM sucrose, 5 μM Lucigenin (GC33485; GlpBio, Montclair, CA, USA), 100 μM NADPH (GC34058; GlpBio, Montclair, CA, USA), and 200 μl mixtures were transferred to 96-cell plates. Luminescence [arbitrary light units (ALUs)] was measured using a microplate reader.

### Mitochondrial ROS detection

Mitochondrial ROS was evaluated by MitoSOX (M36005; Invitrogen, Carlsbad, CA, USA), a specific indicator for mitochondrial ROS. Mito-Track Green (C1048; Beyotime Biotechnology, Shanghai, China) stained mitochondria in live cells and was used for mitochondrial localization. THP-1 macrophages treated as described above were incubated with 2 μM MitoSOX and 50 nM Mito-Track Green for 30 min at 37°C and then analyzed by a confocal laser scanning microscopy.

### Mitochondrial membrane potential detection

Mitochondrial membrane potential (ΔΨm) was measured by a JC-1 probe (M8650; Solarbio Science, Beijing, China) according to the manufacturer’s instructions. JC-1 located on mitochondria under normal conditions is shown in red, while JC-1 appears in green under cell depolarization. THP-1 macrophages treated as described above were stained by JC-1 and subsequently imaged by confocal laser scanning microscopy. The Image software was applied to evaluate fluorescence intensity, and the ratio of red/green indicated the degree of depolarization.

### Mitochondrial Ca^2+^ detection

Mitochondrial Ca^2+^ was assessed with Rhod-2M (40776ES50; Yeasen, Shanghai, China) following manufacturers’ instructions. In brief, THP-1 macrophages treated as described above were incubated with 2 μM Rhod-2M and 50 nM Mito-Track Green for 30 min at 37°C. Then, cells were imaged using confocal laser scanning microscopy.

### Immunofluorescence staining

THP-1 macrophages were seeded at slides and treated as described above. Next, cells were fixed with 4% paraformaldehyde for 20 min and blocked with 1% bovine serum albumin (BSA) containing 0.3% Triton X-100 for 1 h, after which they were incubated with anti-Nrf2 antibody (1:100) overnight at 4°C and subsequently stained with Alexa Fluor 488 goat-anti-mouse IgG for 1 h and sealed with DAPI. The specific fluorescence was imaged using confocal laser scanning microscopy.

### Quantitative real-time PCR

Total RNA was extracted from THP-1 macrophages with TRIzol Reagent (15596026; Thermo Fish, Waltham, MA, US), and RNA was reverse transcribed into cDNA with a Reverse Transcriptase kit (2690S; TakaRa, Tokyo, Japan). Real-time quantitative PCR (RT-qPCR) was performed using a real-time system with SYBR Green Master Mix (3735S; TakaRa, Tokyo, Japan). The β-actin gene was employed as an endogenous control. The relative gene expression levels were analyzed by the 2^−ΔΔCT^ method. The primer sequences (Sangon Biotech, Shanghai, China) are shown in **Supplementary Table S1**.

### Chromatin immunoprecipitation

The interaction between Nrf2 and Klf9 promoter and Klf9 and TXNRD2 promoter was assessed using the ChIP-IT (P2078; Beyotime Biotechnology, Shanghai, China), and qPCR analysis was performed following the manufacturer’s protocol from the kit. The following antibodies were used: control IgG and antibodies specific to Nrf2 and Klf9. The primer sequences (Sangon Biotech, Shanghai, China) were as follows: Klf9 promoter, forward 5’-CGCTAGAGTTACGAAACAGGG-3’; reverse 5’-GAAAGGCCATCCGTTCATGC-3’; TXNRD2 promoter, forward 5′-AACCCTCCCTTCCCAGTTTTG-3′, reverse 5′-AAAAAGCTGGCTCCATGCTG-3′; and GAPDH promoter, forward 5′-TACTAGCGGTTTTACGGGCG-3′, reverse 5′- TCGAACAGGAGCAGAGAGCGA-3′.

### Gene silencing and treatment

THP-1 macrophages were seeded at six-cell plates or slides and then transfected with TXNRD2 siRNA and negative control siRNA (GenePharma, Shanghai, China) using Lipofectamine 2000 (12566014; Invitrogen, Carlsbad, CA, USA) according to manufacturers’ instructions. The specific TXNRD2 siRNA sequence is shown in **Supplementary Table S1**. Then, the cells were primed with 300 ng/ml LPS for 3 h, pretreated with 150 nM LXA4 for 1 h, and subsequently stimulated with 100 μg/ml MSU for 24 h.

### MSU-crystal-induced acute gouty arthritis

Male adult Sprague–Dawley rats (6 weeks, 180–250 g) were purchased from SPF Biotechnology Company (Beijing, China). All the animals were housed in an environment with a temperature of 22 ± 1°C, relative humidity of 50 ± 1%, and a light/dark cycle of 12/12 h. The euthanasia was conducted using mild carbon dioxide (30%) asphyxiation. All animal studies were done in compliance with the regulations and guidelines of Lanzhou University Second Hospital Institutional Animal Care and conducted according to the Association for the Assessment and Accreditation of Laboratory Animal Care (AAALAC) and the Institutional Animal Care and Use Committee (IACUC) guidelines.

The model of gouty arthritis was induced as previously described (10). Rats were then divided into three groups: control group, model group, and LXA4 group. The LXA4 group received 100 μl LXA4 into the ankle joint (10 μg/kg); the same volume of sterile PBS was injected into a rat of the control and model groups. After 1 h, the model and LXA4 groups were injected with 100 μl MSU crystals suspension (500 μg/ml) into the ankle joint, while no treatment was given to the control group. The ankle perimeter of rats was assessed with a string at 12, 24, 48, and 72 h after MSU crystals injection, following which rats were sacrificed. The serum and synovium tissue were collected for further study.

### Histopathological evaluation

The synovium tissue samples were fixed in the 4% paraformaldehyde solution, embedded in paraffin, and sliced into paraffin sections. The sections were stained with H&E as previously described (10). Meanwhile, the synovitis score was estimated as previously described (27, 28).

### Statistical analysis

The data are presented as the mean ± SEM or median with an interquartile range. Statistical analysis was performed using one-way analysis of variance (ANOVA) and subsequent least significant difference (LSD) analysis or signed-rank test or Spearman correlation analysis. *p-*value < 0.05 was considered to be statistically significant.

## Results

### Elevated levels of LXA4 are not enough to fight inflammation in patients with acute gout

**Figure 1:**
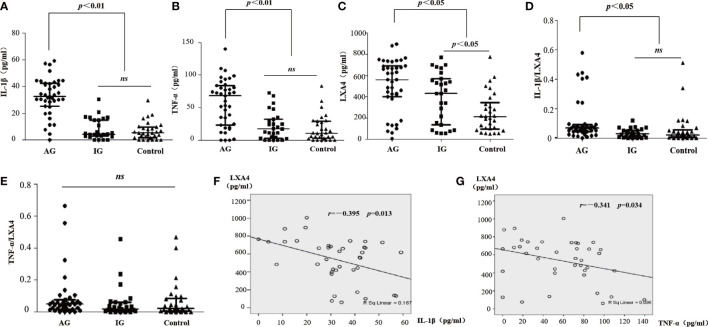
LXA4 was relatively insufficient for the inflammatory response in patients with acute gout. **(A–C)** The levels of IL-1β, TNF-α, and LXA4 in the serum of patients with acute gout (n=39), patients with intercritical gout (n=27), and healthy controls (n=30) detected by ELISA. **(D, E)** The comparison of IL-1β/LXA4 and TNF-α/LXA4 between each group. **(F, G)** The correlation analysis between LXA4 and IL-1β and TNF-α in patients with acute gout. Data are presented as the media with an interquartile range. "ns" is no significant difference.

We detected the levels of LXA4 and inflammatory markers in serum. The levels of IL-1β and TNF-α in patients with AG increased compared with patients with IG and HC (**Figures 1A, B**). We also found that LXA4 was significantly increased in patients with acute gout (**Figure 1C**), and the ratio of IL-1β to LXA4 was significantly increased (**Figures 1D, E**). Moreover, LXA4 showed a significant negative correlation with IL-1β and TNF-α in these patients (**Figure 1F, G**). These results suggest that LXA4 is insufficient to fight inflammatory response in patients with acute gout.

### LXA4 attenuates NLRP3 inflammasome activation induced by MSU crystals

**Figure 2:**
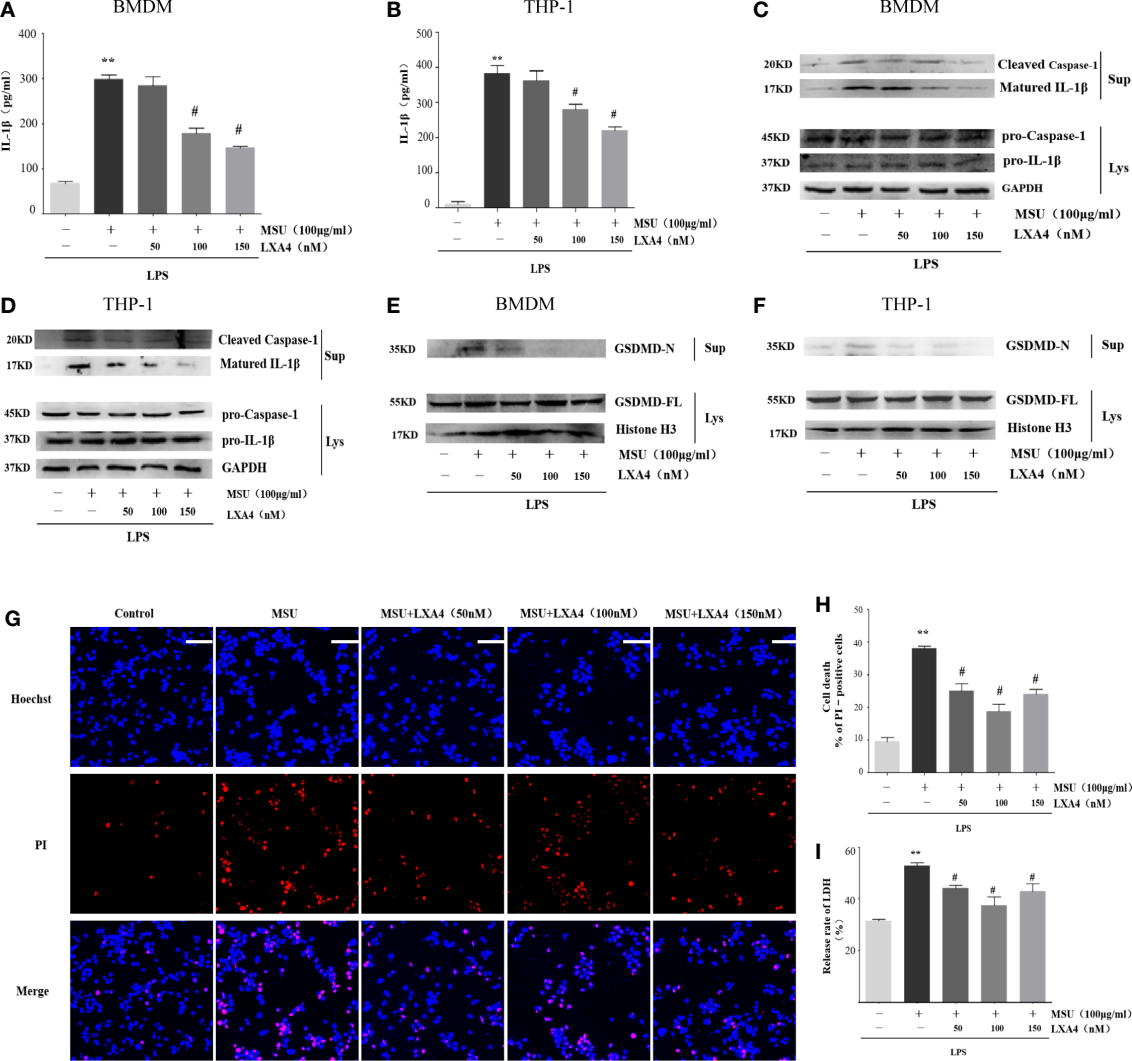
LXA4 attenuates the activation of NLRP3 inflammasome induced by MSU crystals in macrophages. BMDMs and PMA-differentiated THP-1 macrophages were primed with LPS (300 ng/ml) for 3 h and then pretreated with LXA4 (50–150 nM) for 1 h, and subsequently, 100 μg/ml of MSU crystal was added to stimulate for 24 h. **(A, B)** Supernatants were analyzed by ELISA to measure IL-1β levels in BMDMs and PMA-differentiated THP-1 macrophages. **(C–F)** Supernatants and cell lysates were analyzed by immunoblotting to determine the release of cleaved caspase-1 and matured IL-1β and the cleavage of GSDMD-F into GSDMD-N in BMDMs and PMA-differentiated THP-1 macrophages. PMA-differentiated THP-1 macrophages were primed with LPS (300 ng/ml) for 3 h and then pretreated with LXA4 (50-150 nM) for 1 h, and subsequently, 100 μg/ml MSU crystals were added to stimulate for 6 h. **(G)** Cell death was detected by propidium iodide (PI) and Hoechst 33342 staining. Scale bars, 50 µm. **(H)** The percentage of PI-positive cells relative to all cells was calculated in PMA-differentiated THP-1 macrophages. **(I)** The release of LDH in supernatants was detected in PMA-differentiated THP-1 macrophages (n=4). Data are presented as the mean ± SEM, ^**^ *p*<0.01 compared with the vehicle group, ^#^ *p*<0.05 compared with the MSU group.

To explore the intervention potential of LXA4 on NLRP3 inflammasome triggered by MSU crystals in macrophages, we stimulated lipopolysaccharide (LPS)-primed BMDMs and PMA-differentiated THP-1 macrophages with MSU crystals to activate NLRP3 inflammasome, as previously described (29). As shown in **Figures 2A, B**, LXA4 significantly reduced the concentration of IL-1β in macrophages in a dose-dependent manner. The NLRP3 inflammasome activation enhances cleaved caspase-1 manufactured from pro-caspase-1 and matured IL-1β cleaved from pro-IL-1β, and both are released out of the cell. Immunoblotting results further showed elevated cleaved caspase-1 (p20) and matured IL-1β (p17) in the culture supernatants upon MSU crystals stimulation. This process was reversed by LXA4 (**Figures 2C, D**). Yet, LXA4 did not inhibit the precursors of IL-1β and caspase-1 protein expression (**Figures 2C, D**). These findings indicate that LXA4 may interfere with the activation signal rather than the priming signal of NLRP3 inflammasome.

Besides promoting IL-1β secretion, the activation of NLRP3 inflammasome can cause inflammatory death to cells, especially NLRP3-mediated pyroptosis (7, 10, 11, 30, 31). Immunoblotting results showed that the MSU crystal enhances the GSDMD-N in the cell supernatant of macrophages, the execution protein for pyroptosis (32), while LXA4 attenuates the elevated GSDMD-N (**Figures 2E, F**).

Next, we adopted propidium iodide (PI) uptake (red fluorescence) and cellular LDH release to evaluate pyroptosis directly. LXA4 prevented the PI staining (**Figures 2G, H**) and decreased the cellular LDH release of differentiated THP-1 macrophages (**Figure 2I**). Taken together, we concluded that LXA4 attenuates the MSU-crystal-induced NLRP3 inflammasome activation in macrophages.

### LXA4 antagonizes the assemble of NLRP3 inflammasome

**Figure 3:**
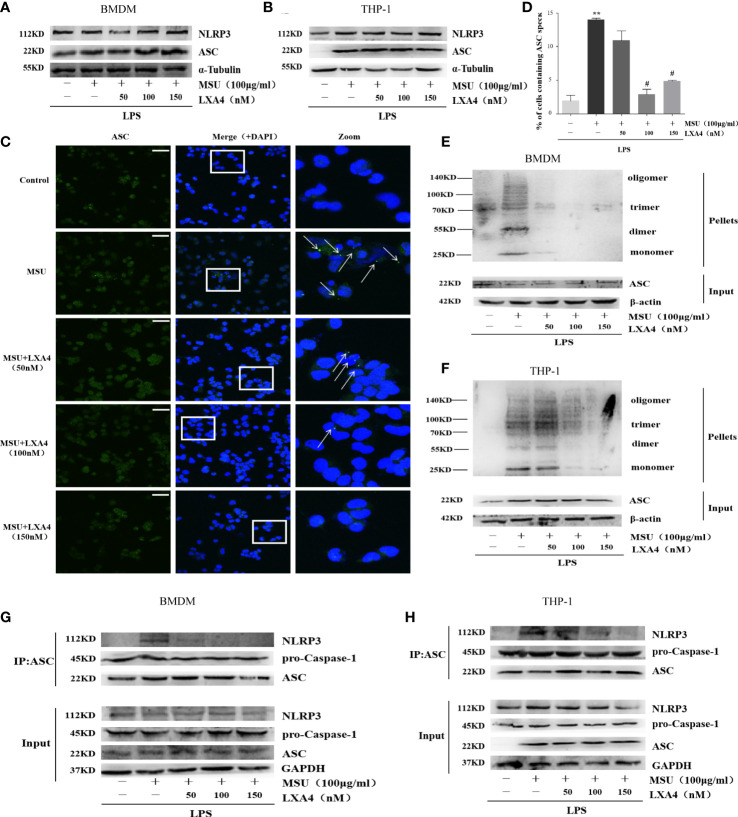
LXA4 antagonizes the assembly of NLRP3 inflammasome. BMDMs and PMA-differentiated THP-1 macrophages were primed with LPS (300 ng/ml) for 3 h and then pretreated with LXA4 (50–150 nM) for 1 h; subsequently, 100 μg/ml of MSU crystals was added for 24 h. **(A, B)** Cell lysates were analyzed by immunoblotting to detect NLRP3 and ASC protein levels. **(C)** ASC speck formation was determined by immunofluorescence images in PMA-differentiated THP-1 macrophages; green shows ASC, blue shows nuclei; scale bars, 20 µm. **(D)** The percentage of cells with ASC-speck relative to all cells (n=3). **(E, F)** ASC oligomerization in pellets cross-linked with DSS was detected by immunoblotting in BMDMs and PMA-differentiated THP-1 macrophages. **(G, H)** The interaction between ASC and NLRP3, and pro-caspase-1 was analyzed by immunoprecipitation using an antibody against ASC and then measured by immunoblotting in BMDMs and PMA-differentiated THP-1 macrophages. Data are presented as the mean ± SEM, ^**^ *p*<0.01 compared with the vehicle group, ^#^ *p*<0.05 compared with the MSU group.

Next, we sought to investigate the mechanisms through which LXA4 attenuates the NLRP3 inflammasome activation induced by MSU crystals. We observed that LXA4 barely affects NLRP3 and ASC expression consistent with pro-caspase-1 in macrophages (**Figures 3A, B**), suggesting again that LXA4 may affect the assembly but not the expression of NLRP3 inflammasome. The formation of perinuclear structures called ASC speck and oligomerization of ASC are indispensable for assembling and activating the NLRP3 inflammasome (31, 33). Our results found that LXA4 significantly reduced the formation of ASC specks induced by MSU crystal in differentiated THP-1 macrophages (**Figures 3C, D**). Furthermore, we used DSS to cross-link ASC and investigated ASC oligomerization by immunoblotting and found a similar result that LXA4 markedly repressed ASC oligomerization in macrophages (**Figures 3E, F**).

The premise of NLRP3 inflammasome activation is that the components are completely assembled (34). Herein, we employed co-immunoprecipitation to analyze the interaction between ASC with NLRP3 and pro-caspase-1. ASC-NLRP3 interaction triggered by MSU crystal was markedly reduced by LXA4 in macrophages (**Figures 3G, H**); yet, LXA4 had no effect on the ASC-pro-caspase-1 interaction (**Figures 3G, H**). It has been reported that the interaction between NLRP3 and NIMA-related kinase 7 (NEK7) enhances inflammasome activation (11, 35). However, in our study, LXA4 did not affect the expression of NEK7 (**Supplementary Figures S1A, C**) or NEK7–NLRP3 interaction (**Supplementary Figures S1B, D**). These data suggest that LXA4 antagonizes the assembly of the NLRP3 inflammasome by impeding the specks formation and oligomerization of ASC and the ASC-NLRP3 interaction.

### LXA4 inhibits ROS production rather than affects chloride efflux and potassium efflux

**Figure 4:**
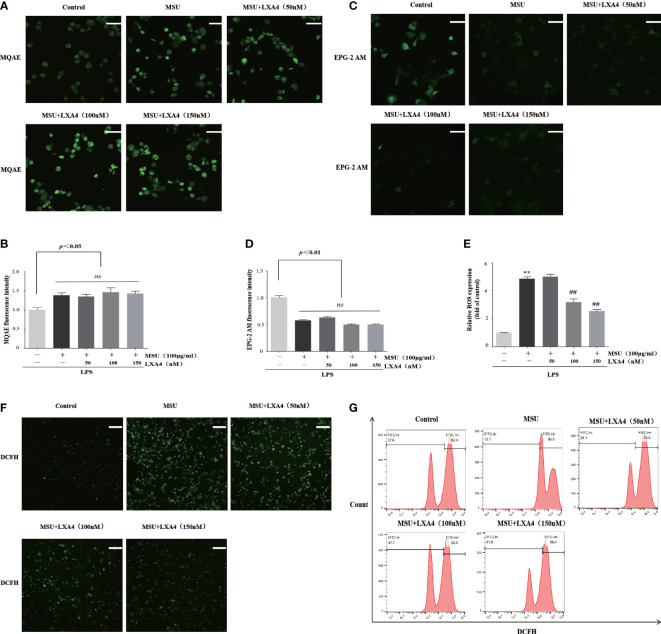
LXA4 inhibits ROS production rather than affects chloride efflux and potassium efflux. The PMA-differentiated THP-1 macrophages were primed with LPS (300 ng/ml) for 3 h and then pretreated with LXA4 (50–150 nM) for 1 h; subsequently, 100 μg/ml of MSU crystals was added for 24 h. **(A, B)** Cells were incubated with MQAE to measure intracellular chloride ions and compare relative fluorescence intensity. The higher fluorescence intensity indicates a lower level of chloride ions and *vice versa*. Scale bars, 20 µm. The relative fluorescence intensity of MQAE was calculated (n=3). **(C, D)** Cells incubated with EPG-2 AM to measure intracellular potassium ions and compare relative fluorescence intensity (n=3). Scale bars, 20 µm. The relative fluorescence intensity of EPG-2AM (n=3). **(E–G)** Cells were stained with DCFH (10 μM) and then analyzed by a microplate reader (n=3), florescent microscope, and flow cytometry. Scale bars, 50 µm. Data are presented as the mean ± SEM, ^**^ *p*<0.01 compared with the vehicle group, ^##^ *p*<0.01 compared with the MSU group. "ns" is no significant difference.

We next determined whether LXA4 can hamper the upstream event dependent on NLRP3 inflammasome activation. Previous studies have confirmed that chloride efflux, potassium efflux, and ROS production are important upstream events for activation of NLRP3 inflammasome (15, 16, 36, 37). The MQAE is a fluorescence probe for intracellular chloride ions; the higher intensity of MQAE fluorescence shows a lower level of intracellular chloride ions and *vice versa*. The MQAE data revealed that the MSU crystal triggered a marked decrease in chloride efflux; nevertheless, LXA4 did not improve this decrease (**Figures 4A, B**). Consistently, EPG-2M data (a fluorescence probe for intracellular potassium) showed that LXA4 had no effect on the potassium efflux induced by MSU crystal (**Figures 4C, D**).

Furthermore, we used DCFH (a ROS fluorescence probe) to detect ROS levels. The results from fluorescence microscopy, microplate reader, and flow cytometry analysis demonstrated that MSU crystal resulted in a significant increase in ROS; yet, this process was reversed by LXA4 (**Figures 4E–G**), suggesting that LXA4 suppressed the MSU-crystal-triggered ROS generation. We speculate that LXA4 attenuates NLRP3 inflammasome activation induced by MSU crystal, probably through blocking ROS production.

### LXA4 blunts the activation of NADPH oxidase and the dysfunction of mitochondria

**Figure 5:**
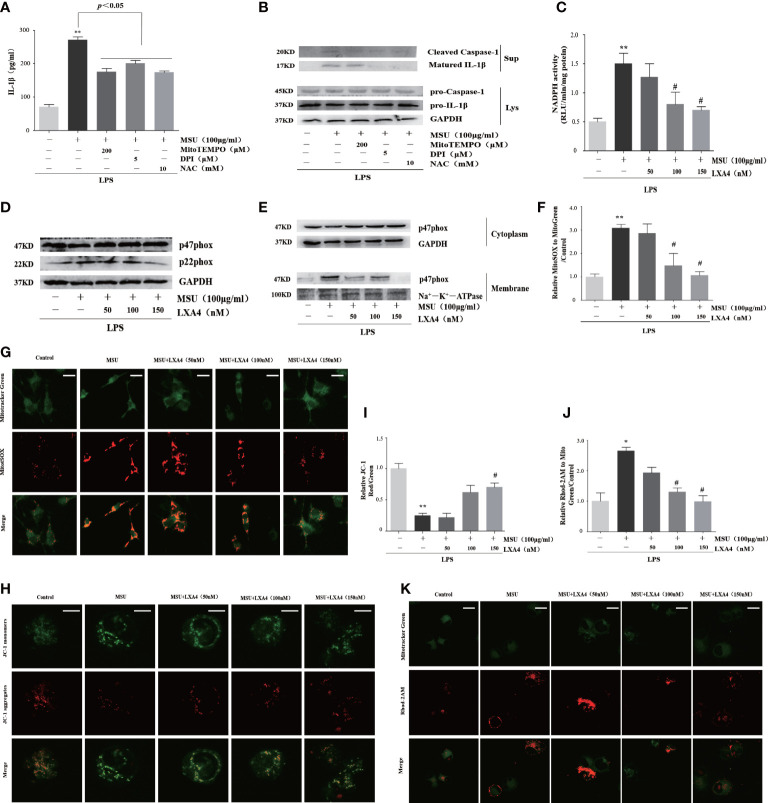
LXA4 blunts the activation of NADPH oxidase and the dysfunction of mitochondria. **(A, B)** The PMA-differentiated THP-1 macrophages were primed with LPS (300 ng/ml) for 3 h and then pretreated with NAC (10 mM), MitoTEMPO (200 μM), and DPI (5 μM); then, 100 μg/ml of MSU crystals was added for 24 h. **(A)** Supernatants were analyzed by ELISA to measure IL-1β (n=3). **(B)** Supernatants and cell lysates were analyzed by immunoblotting to determine the release of cleaved caspase-1 and matured IL-1β. The PMA-differentiated THP-1 macrophages were primed with LPS (300 ng/ml) for 3 h and then pretreated with LXA4 (50–150 nM) for 1 h; subsequently, 100 μg/ml of MSU crystals was added for 24 h. **(C)** The NADPH oxidase activity was analyzed by lucigenin-enhanced chemiluminescence (n=3). **(D)** Cell lysates were analyzed by immunoblotting to determine the p22phox and p47phox protein levels. **(E)** The cytosol and membrane lysates were analyzed by immunoblotting to determine the p47phox protein levels. **(F, G)** The THP-1 macrophages were stained with MitoSOX (red) and MitoTracker (green) to determine mitochondrial ROS levels. Scale bars, 10 µm. The relative fluorescence intensity of MitoSOX to MitoTracker was calculated (n=3). **(H, I)** The THP-1 macrophages were stained with JC-1 to measure mitochondrial ΔΨm, and the ΔΨm were calculated by the ratio of red/green (n=3). Scale bars, 5 µm. **(J, K)** THP-1 macrophages were stained with Rhod-2M (Red) and MitoTracker (Green) to determine mitochondrial *Ca^2+^* levels. Scale bars, 10 µm. The *Ca^2+^* levels were calculated by the ratio of Rhod-2M/Mito Green (n=3). Data are presented as the mean ± SEM, ^*^ *p*<0.05 and ^**^ *p*<0.01 compared with the vehicle group, ^#^ *p*<0.05 compared with the MSU group.

We further studied the effect of LXA4 on the source of ROS. To inquire about the source of ROS in our experimental model, we pretreated cells with inhibitors of ROS, including NAC (a cellular ROS scavenger), MitoTEMPO (a mitochondrial ROS scavenger), and DPI (a specific inhibitor for NADPH oxidase). As shown in **Figures 5A, B**, all inhibitors could depress the release of cleaved caspase-1 and matured IL-1β, indicating that ROS from activated NADPH oxidase and damaged mitochondria are the mediator for the NLRP3 inflammasome activation induced by MSU crystal, which is consistent with other studies (13, 38, 39). Accordingly, we further examined whether LXA4 could prevent NADPH oxidase activation and mitochondria dysfunction. We found that the MSU-crystal-triggered NADPH oxidase activation was markedly reduced by LXA4 (**Figure 5C**).

p47phox and p22phox are the essential cytoplasmic and membrane subunit of NADPH oxidase (40). We found that pretreatment with LXA4 hardly affects the p47phox but decreases the p22phox protein level (**Figure 5D**). Moreover, MSU crystal decreased p47phox in cytosol lysates and increased p47phox in membrane lysates, but LXA4 overturned the changes (**Figure 5E**), indicating that LXA4 hampers the transfer of p47phox from the cytoplasm to the membrane.

Meanwhile, we tested the mitochondrial ROS generation by mitochondrial ROS-specific indicator MitoSOX staining and found that MSU-crystal-triggered ROS augmentation in mitochondria was reduced by LXA4 (**Figures 5F, G**). The depolarization of membrane potential and grave Ca^2+^ overload are the inducements and signs of serious mitochondrial damage (41, 42). Using the specific indicator, we found that LXA4 relieved the depolarization of the membrane potential and Ca^2+^ overload in mitochondria at least partly (**Figures 5H–K**). We propose that LXA4 blunts MSU-crystal-induced ROS generation due to the suppression of NADPH oxidase activation and improvement of mitochondrial dysfunction.

### LXA4 suppress Nrf2 activation

**Figure 6:**
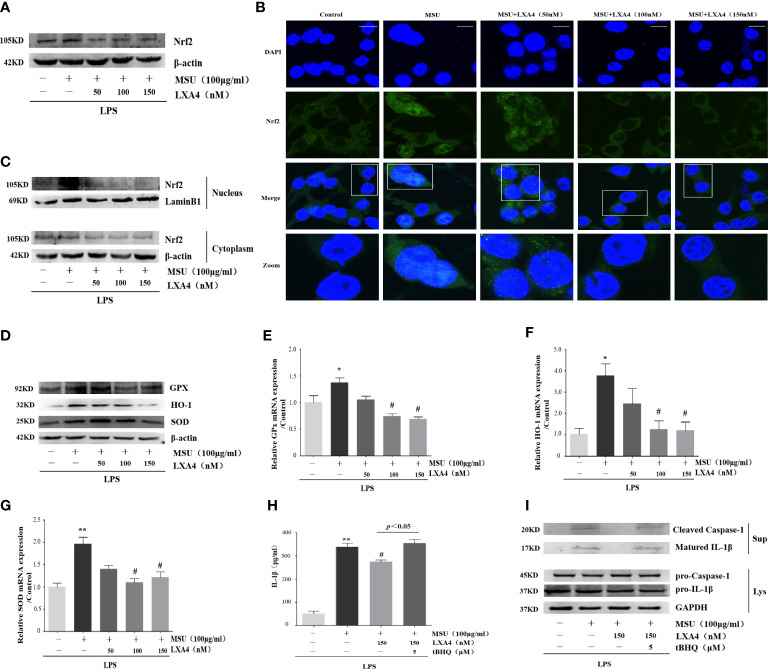
LXA4 suppresses Nrf2 activation. The PMA differentiated THP-1 macrophages were primed with LPS (300 ng/ml) for 3 h and then pretreated with LXA4 (50-150 nM) for 1 h; subsequently, 100 μg/ml of MSU crystals was added for 24 h. **(A)** Cell lysates were analyzed by immunoblotting to determine the Nrf2. **(B)** The nuclear location of Nrf2 was visualized by immunofluorescence analysis with an anti-Nrf2 antibody. Scale bars, 20 µm. **(C)** The cytoplasmic and nuclear lysates were analyzed by immunoblotting to determine the Nrf2. **(D)** Cell lysates were analyzed by immunoblotting to determine the HO-1, SOD, and GPx protein levels. **(E–G)** RT-qPCR was performed to measure the HO-1, SOD, and GPx mRNA levels (n=4). The PMA-differentiated THP-1 macrophages were primed with LPS (300 ng/ml) for 3 h and then pretreated with LXA4 (150 nM) and tBHQ (5 μM) for 1 h; subsequently, 100 μg/ml MSU crystals were added to stimulate for 24 h. **(H)** Supernatants were analyzed by ELISA to measure IL-1β (n=4). **(I)** Supernatants and cell lysates were analyzed by immunoblotting to determine the release of cleaved caspase-1 and matured IL-1β. Data are presented as the mean ± SEM, ^*^ *p*<0.05 and ^**^ *p*<0.01 compared with the vehicle group, ^#^ *p*<0.05 compared with the MSU group.

Nrf2 is a classical and pivotal regulator with an antioxidant role (43–45). LXA4 can arouse Nrf2 activation to mediate antioxidant and anti-inflammatory activity (17–19). Additionally, MSU evoking IL-1β/NLRP3 inflammasome is dependent of Nrf2 activation (11, 20). Consequently, we wondered whether LXA4 could regulate Nrf2 signaling. Consistent with previous reports (11, 20), our results revealed that the MSU crystal increased Nrf2 in differentiated THP-1 macrophages; however, LXA4 mitigated the protein level of Nrf2 (**Figure 6A**). The immunofluorescence analysis further showed that LXA4 relieved the nuclear translocation of Nrf2 triggered by the MSU crystal (**Figure 6B**). Simultaneously, immunoblotting results revealed that LXA4 rectified the MSU-crystal-caused increase in Nrf2 in nuclear lysates (**Figure 6C**), suggesting that LXA4 prevents the nuclear movement of Nrf2. Interestingly, the antioxidants encoded by Nrf2, including HO-1, SOD, and GPx and their mRNA and protein expression were strongly depressed by LXA4 (**Figures 6D–G**). These results indicate that LXA4 inhibits the activation of Nrf2.

Moreover, we adopted tBHQ, an activator for Nrf2, to resume the diminished Nrf2 caused by LXA4. We found that the LXA4-induced depression in the release of cleaved caspase-1 and matured IL-1β was reversed by tBHQ (**Figures 6H, I**). These results indicate that LXA4 prevents MSU-crystal-triggered NLRP3 inflammasome activation *via* suppressing Nrf2.

### LXA4 interdicts Nrf2/Klf9/TXNRD2 signaling pathway

**Figure 7:**
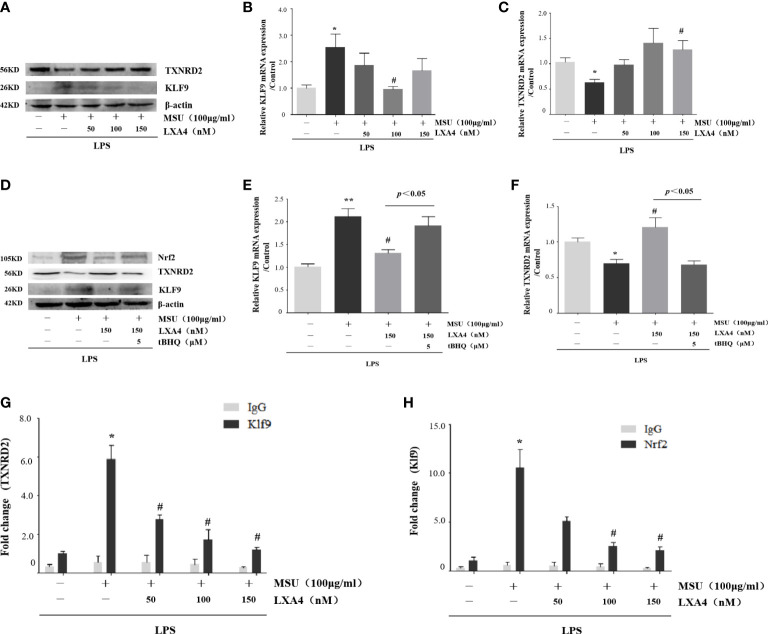
LXA4 interdicts Nrf2/Klf9/TXNRD2 signaling pathway. The PMA-differentiated THP-1 macrophages were primed with LPS (300 ng/ml) for 3 h and then pretreated with LXA4 (50–150 nM) for 1 h; subsequently, 100 μg/ml of MSU crystals was added for 24 h. **(A)** Cell lysates were analyzed by immunoblotting to determine the Klf9 and TXNRD2 protein levels. **(B, C)** RT-qPCR was performed to measure the TXNRD2 and Klf9 mRNA levels (n=5). The PMA-differentiated THP-1 macrophages were primed with LPS (300 ng/ml) for 3 h and then pretreated with LXA4 (150 nM) and tBHQ (5 μM) for 1 h; subsequently, 100 μg/ml of MSU crystals were added for 24 h. **(D)** Cell lysates were analyzed by immunoblotting to determine the Nrf2, Klf9, and TXNRD2 protein levels. **(E, F)** RT-qPCR was performed to measure the TXNRD2 and Klf9 mRNA levels (n=4). The PMA-differentiated THP-1 macrophages were primed with LPS (300 ng/ml) for 3 h and then pretreated with LXA4 (50–150 nM) for 1 h; subsequently, 100 μg/ml of MSU crystals was added to stimulate for 24 h. **(G, H)** The chromatin was immunoprecipitated with control (IgG) or Nrf2- and Klf9-specific antibody, followed by the reversal of the cross-linking and DNA isolation. DNA was used in quantitative PCR with primers specific for Klf9 and TXNRD2 promoter or irrelevant GAPDH promoter (n=4). Data are presented as the mean ± SEM, ^*^ *p*<0.05 and ^**^ *p*<0.01 compared with the vehicle group, ^#^ *p*<0.05 compared with the MSU group.

As LXA4 inhibited Nrf2 and antioxidants encoded by Nrf2, we turned our attention to the Klf9/TXNRD2 axis; still, a signaling pathway mediated by Nrf2 was involved in oxidative stress (46). As shown in **Figures 7A–C**, MSU crystal increased Klf9 and decreased TXNRD2 (both protein and mRNA levels), while LXA4 reversed this process. To validate the regulation of LXA4 on the Nrf2/Klf9/TXNRD2 axis, we adopted tBHQ to resume the diminished Nrf2 caused by LXA4. We observed that tBHQ overthrows the inhibitory effect of LXA4 on Klf9 and the promotional effect of LXA4 on TXNRD2 (**Figures 7D–F**). These results suggest that LXA4 interdicts the Nrf2/Klf9/TXNRD2 signaling pathway.

Given that Nrf2 can occupy the promoter of Klf9 and Klf9 can occupy the promoter of TXNRD2 (46), we performed a ChIP assay to determine whether LXA4-induced repression of Klf9 and promotion of TXNRD2 are associated with a decrease in these occupancies. Results showed that a significant enrichment of Klf9- and TXNRD2-promoter-specific DNA was detected in the chromatin material precipitated with Nrf2- and Klf9-specific antibodies in THP-1 macrophages induced by the MSU crystal, respectively; however, LXA4 depressed both (**Figures 7G, H**), indicating that LXA4 disturbs the interaction between Nrf2 and the promoter of Klf9, and Klf9 and the promoter of TXNRD2. These findings further confirm the regulation of LXA4 on the Nrf2/Klf9/TXNRD2 pathway.

### LXA4 alleviates the MSU-crystal-triggered NLRP3 inflammasome activation through a TXNRD2-dependent manner

**Figure 8:**
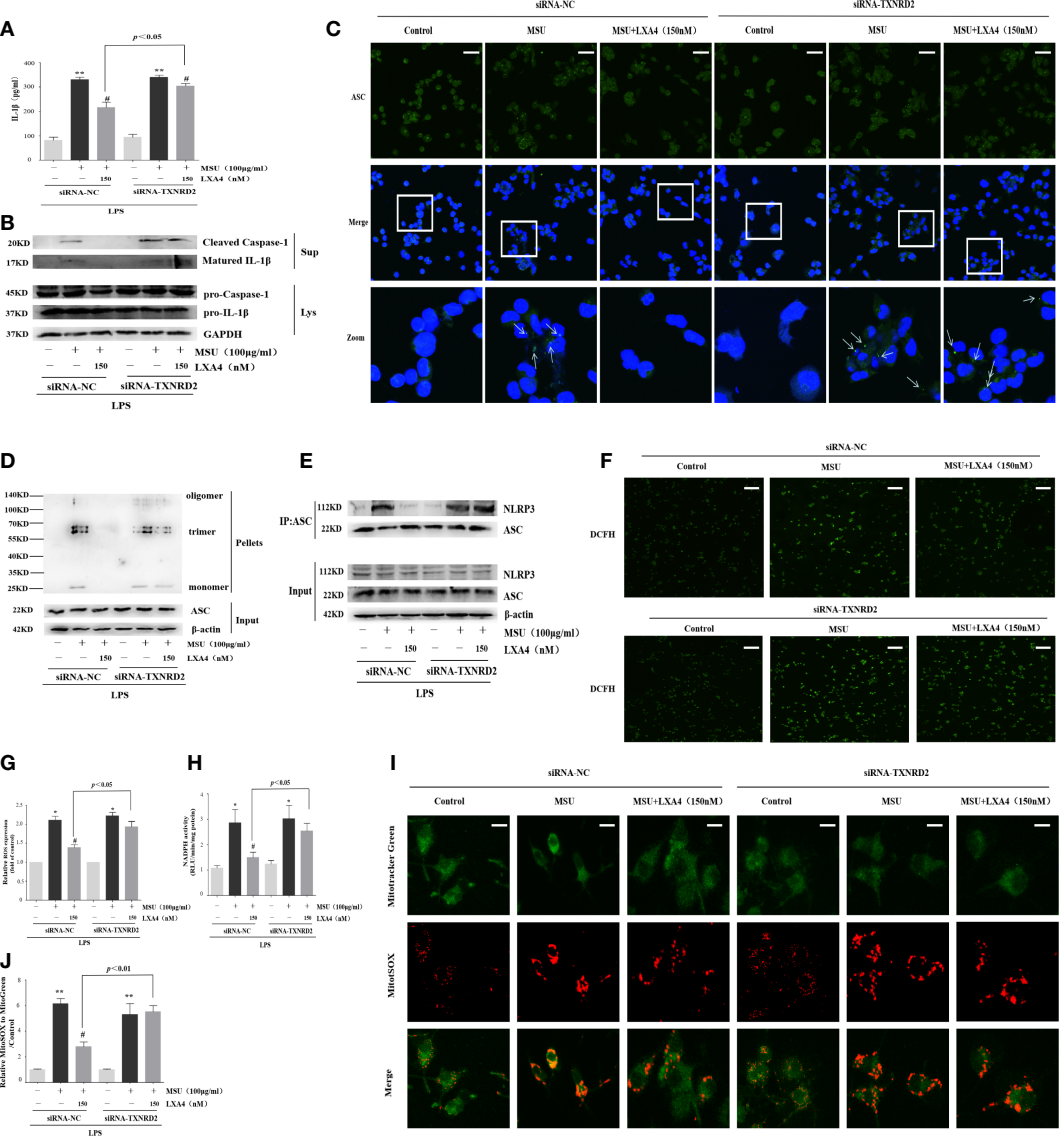
LXA4 alleviates the MSU-crystal-triggered-NLRP3 inflammasome activation through a TXNRD2-dependent manner. The PMA-differentiated THP-1 macrophages were transfected with TXNRD2 siRNA before being primed with LPS and then pretreated with LXA4 (150 nM) for 1 h; subsequently, 100 μg/ml of MSU crystals were added for 24 h. **(A)** Supernatants were analyzed by ELISA to measure IL-1β (n=3). **(B)** Supernatants and cell lysates were analyzed by immunoblotting to determine the release of cleaved caspase-1 and matured IL-1β. **(C)** ASC-speck formation was determined by immunofluorescence image. Scale bars, 20 µm. **(D)** ASC oligomerization in pellets. **(E)** The interaction between ASC and NLRP3. **(F, G)** The ROS levels were analyzed by a florescent microscope and microplate reader (n=3). Scale bars, 50 µm. **(H)** The NADPH oxidase activity was analyzed by lucigenin-enhanced chemiluminescence (n=4). **(I)** The mitochondrial ROS levels were detected by MitoSOX. Scale bars, 10 µm. **(J)** The relative fluorescence intensity of MitoSOX to MitoTracker was calculated (n=3). Data are presented as the mean ± SEM, ^*^ *p*<0.05 and ^**^ *p*<0.01 compared with the vehicle group, ^#^ *p*<0.05 compared with the MSU group.

TXNRD2 is a mediator endowed with antioxidant ability and located on the Nrf2/Klf9/TXNRD2 axis terminal. Given that the above results suggested that LXA4 could elevate TXNRD2 expression by suppressing Nrf2, we further explored whether LXA4-induced attenuation of the MSU-crystal-triggered NLRP3 inflammasome activation is dependent on TXNRD2. To address this, we employed a TXNRD2 silence strategy and established TXNRD2-depleted cells by small interfering RNA in differentiated THP-1 macrophages (**Supplementary Figures S2A, B**). The results showed that siRNA-TXNRD2 could block the inhibitory impact of LXA4 on the IL-1β release, specks formation, and oligomerization of ASC, and the ASC-NLRP3 interaction (**Figures 8A–E**, **Supplementary Figure S3**). Similarly, auranofin, a specific inhibitor for TXNRD2, also neutralized LXA4-induced inhibition of the IL-1β release (**Supplementary Figures S4A, B**). Interestingly, we observed that siRNA-TXNRD2 also impaired the inhibitory influence of LXA4 on superior total ROS generation (**Figures 8F, G**). In addition, siRNA-TXNRD2 reversed the suppressive action of LXA4 on NADPH oxidase activation and mitochondria ROS production (**Figures 8H–J**). We conclude that LXA4 interferes with ROS generation to prevent NLRP3 inflammasome activation dependent on TXNRD2.

### LXA4 mitigates the severity of MSU-crystal-induced gouty arthritis in a mouse model

**Figure 9:**
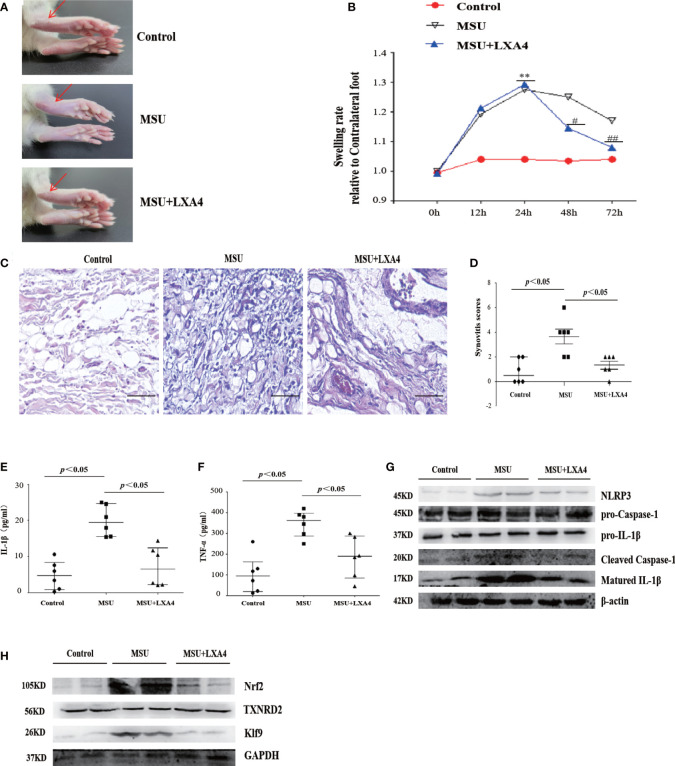
LXA4 attenuates the severity of MSU-crystal-induced gouty arthritis in a mouse model. SD rats were treated with an intraarticular injection of MSU crystals (100 μl, 500 μg/ml) for 72 h in the presence of LXA4 (10 μg/kg/rat). **(A, B)** Representative photographs to present the swelling of joints and swelling paw index at different time points (n=6). **(C, D)** Hematoxylin and eosin (H&E)-stained infiltrated leukocytes in joint tissues and synovitis score measurement (n=6). Scale bars, 100 μm. **(E, F)** The levels of IL-1β and TNF-α in the serum of control rats, gouty arthritis rats, and LXA4+gouty arthritis rats. **(G)** The NLRP3, pro-caspase-1, pro-IL-1β, cleaved caspase-1, and matured IL-1β in joint tissues were analyzed by immunoblotting. **(H)** The Nrf2, Klf9, and TXNRD2 in joint tissues were analyzed by immunoblotting. Data are presented as the mean ± SEM, or media with interquartile range; ^**^ *p*<0.01 compared with the vehicle group, ^#^ *p*<0.05 and ^##^ *p*<0.01 compared with the MSU group.

To investigate the therapeutic potential of LXA4 for gout *in vivo*, we established a gouty arthritis rat model. Our results showed that LXA4 markedly relieved acute joint swelling of gouty arthritis rats (**Figures 9A, B**). Histological analysis further showed that LXA4 inhibited the massive infiltration of inflammatory cells in joint tissues (**Figures 9C, D**). In addition, LXA4 could significantly depress serum IL-1β and TNF-α in gouty arthritis rats (**Figures 9E, F**). Interestingly, LXA4 also significantly inhibited the expression of NLRP3, cleaved caspase-1, and matured IL-1β in the joint of gouty arthritis rats (**Figure 9G**).

To validate the regulatory effect of LXA4 on the Nrf2/Klf9/TXNRD2 signaling pathway, we detected the key protein of this pathway in a rat model. As shown in **Figure 9H**, LXA4 also subverted the increase in Nrf2 and Klf9 and depression of TXNRD2 in gouty arthritis rats, which is consistent with *in vitro* data. Thus, these results approve that LXA4 may be a potential candidate in the treatment of gouty arthritis.

## Discussion

The current study demonstrated that LXA4 is a reliable inhibitor for the NLRP3 inflammasome stimulated by MSU crystals. We used LXA4 as a jammer and found that LXA4 could attenuate MSU-crystal-activated NLRP3 inflammasome in BMDM and THP-1 macrophages. Furthermore, LXA4 also alleviated the severity of NLRP3-driven inflammation in the MSU-crystal-induced gouty arthritis mouse model. Mechanistically, LXA4 reduced ROS generation by suppressing Nrf2 to promote TXNRD2, thereby inhibiting NLRP3 inflammasome activation induced by MSU crystal. Therefore, our findings identified a previously undescribed regulatory mechanism whereby LXA4 prevented NLRP3 inflammasome activation by suppressing Nrf2.

LXA4 is an endogenous lipoxygenase-derived eicosanoid mediator that has been associated with fewer drug side effects and strong anti-inflammation properties (17–19). The alteration of LXA4 in our body may be responsible for some diseases. In this study, we found higher levels of LXA4 in acute gout compared with healthy controls; however, the levels of IL-1β were significantly elevated. Previous studies have reported that LXA4 is increased in some inflammatory diseases (47, 48). As an endogenous anti-inflammatory factor, the body can exert some self-protection roles by releasing LXA4 when stimulated by harmful stimulus (47), so the levels of LXA4 were increased in acute gout. However, as the levels of LXA4 released from our body are not enough to defeat inflammatory responses induced by harmful stimuli, the levels of IL-1β in acute gout are elevated.

The treatment with LXA4 has been reported to inhibit NLRP3 inflammasome activation by diverse roles. LXA4 can suppress autophagy-related pathways, the JNK1/Beclin-1/PI3KC3 axis, to reduce NLRP3 activation in non-compressive disk herniation (49). In a model of osteoclast-mediated diabetic osteoporosis, LXA4 inhibits NLRP3-inflammasome-mediated IL-1β and IL-18 production by fading the nuclear factor kappa B (NF-κB) pathway (50). Our *in vitro* data indicated that LXA4 reduces the activation of NLRP3 inflammasome induced by MSU crystals in macrophages and depresses IL-1β secretion and pyroptosis (**Figure 2**). Two-step signals are required to activate the NLRP3 inflammasome (7, 12). In the present study, we demonstrated that LXA4 did not suppress the expression of NLRP3, ASC, pro-IL-1β, and pro-caspase-1. Therefore, LXA4 is ineffective on the first signal of NLRP3 activation. As the second signal, MSU crystals master the assembly of the NLRP3 inflammasome, which leads to the consequence that pro-IL-1β and pro-caspase-1 are manufactured to cleaved caspase-1 and matured IL-1β, respectively (10, 11). Notably, ASC is indispensable for activating NLRP3 inflammasome and subsequent maturation of IL-1β (7). Our results also showed that the MSU-crystal-triggered ASC specks, ASC oligomerization, and ASC-NLRP3 interaction were attenuated by LXA4 (**Figure 3**). These results suggest that LXA4 reduces the NLRP3 inflammasome activation induced by MSU crystal as a result of hampering the assembly of the NLRP3 inflammasome.

Abnormal ROS production is a critical upstream event for triggering NLRP3 inflammasome assembly (12). It has been widely confirmed that superior ROS are the pivotal regulator for the NLRP3 inflammasome activation induced by the MSU crystal (5, 10, 11, 30). Recent studies have found that ROS inhibition reversibly affects MSU-crystal-induced inflammatory responses (14, 16). LXA4 has a powerful antioxidant activity and can exert its anti-inflammatory role by hampering oxidative stress. Previous studies have found that LXA4 prevents the expression of inflammatory factors, such as TNF-α, IL-1β, IL-6, and inflammasome activation, by inhibiting ROS in different inflammatory damages (17–19). In this study, we demonstrated that MSU-crystal-triggered excessive intracellular ROS was reduced by LXA4 (**Figure 5**). The MSU-crystal-triggered inflammatory response is mediated by damaged mitochondria and activated NADPH oxidase, acting as a source of ROS (13, 38, 39). Furthermore, we found that LXA4 depresses the NADPH oxidase activation and improves mitochondrial dysfunction. To sum up, LXA4 antagonizes the activation of NLRP3 inflammasome induced by MSU crystal by impeding ROS generation.

Nrf2 has a crucial protective role in inflammation under oxidative stress and is a widely recognized antioxidant butler (21). The activated Nrf2 can promote antioxidant protein expression and trigger related antioxidant systems to exert its antioxidant function (43, 44, 51). The activation of Nrf2 is considered the chief measure of some cell protectors developing antioxidant effects and subsequent anti-inflammatory roles (51). Previous studies have found that LXA4 alleviates inflammation by activating Nrf2 in acute lung injury, ischemia–reperfusion injury, and spinal cord injury (17–19). However, Nrf2 is required for NLRP3 inflammasome activation induced by stimulus, including MSU crystal, ATP, and nigericin (9, 20, 52). A recent study reported that increasing Nrf2 level after MSU crystal stimulation could weaken ROS production and subsequent NLRP3 inflammasome activation (11, 20). Surprisingly, here, we provided interesting evidence that LXA4 inhibited the activation of Nrf2 and expression of Nrf2-mediated classical antioxidant protein, including HO-1, SOD, and GPX (**Figures 6A–F**). Moreover, resuming Nrf2 by tBHQ disturbed the LXA4-induced depression of NLRP3 inflammasome activation (**Figures 6H, I**). To the best of our knowledge, no study reported that cytoprotective agents exerted their antioxidant and anti-inflammatory function by suppressing Nrf2 (13, 18, 19, 53). These surprising results suggested that LXA4 did not employ the Nrf2 pathway but suppressed Nrf2 to exert a biological role in our experimental model. Thus, we plan to further explore how LXA4 regulates the pathway involving Nrf2 inhibition to oppose MSU-crystal-induced NLRP3 inflammasome.

The Nrf2/Klf9/TXNRD2 axis is a signaling pathway involved in oxidative stress mediated by Nrf2 (46). Klf9 is an upregulated target gene for Nrf2 and initiator of cellular oxidative stress when the cell is faced with a lethal stimulus (46, 54). Klf9 depresses multiple antioxidant defense genes, including TXNRD2, to enhance cellular ROS and oxidant-dependent cell damage (21, 55). Since Klf9 depletion can also relieve oxidative stress, Klf9 downregulation may protect inflammatory response by promoting TXNRD2 expression (46). Our results showed that LXA4 reduces the expression of Klf9 and enhances the expression of TXNRD2 at mRNA and protein levels. Yet, this process was reversed when the activator of Nrf2 was applied (**Figure 7**). In addition, LXA4 hampered the interaction between Nrf2 and the promoter of Klf9, and Klf9 and the promoter of TXNRD2. Thus, we concluded that the Klf9/TXNRD2 axis is modulated when LXA4 suppresses Nrf2 activation. As an ROS-detoxifying enzyme, TXNRD2 is critical for cellular redox homeostasis, and TXNRD2 deficiency can contribute to oxidative damage in myeloma cells (55), endothelium (56), and myocardial ischemia/reperfusion mice (57). Overexpression of TXNRD2 can ameliorate multiple oxygen metabolic processes and lessen ROS generation, thereby attenuating the downstream inflammatory response (58). Herein, we observed that TXNRD2 knockdown reversed the inhibitory effect of LXA4 on the NLRP3 inflammasome activation and ROS generation (**Figure 8**). Taken together, these data suggest that LXA4 prevents NLRP3 inflammasome activation by MSU crystal through a TXNRD2-dependent manner.

Our results showed that LXA4 could attenuate inflammation of joints and inflammatory cytokines levels in serum in MSU-crystal-triggered gouty arthritis. *In vitro* data preliminarily identified the potential of LXA4 to treat gouty arthritis. IL-1β induced by MSU crystal promotes the recruitment of inflammatory cells, especially neutrophils, at the sites of inflammation, and triggers gouty inflammation, which is considered an important pathological hallmark of gout. Furthermore, our *in vivo* results showed that LXA4 inhibits massive infiltration of neutrophils in joint tissues of the gouty arthritis model. Neutrophils have NLRP3 inflammasome, and their activation is an indispensable process for the sensitization and infiltration of neutrophils. In this study, we found that LXA4 could inhibit NLRP3 inflammasome in gouty arthritis model, which is consistent with other studies (59, 60). However, it remains unclear why LXA4 did not affect the expression of NLRP3 *in vitro*, while it inhibited NLRP3 production *in vivo*. On the one hand, in animal model lakes, the first signal for NLRP3 inflammasome activation like LPS, and MSU can enhance NLRP3 expression by the NF-κB pathway *in vivo* (61). On the other hand, there is a complex internal environment in the animal model compared to cell experiments. Gout is regulated by multiple pathways induced by the MSU crystal, so NLRP3 production is upregulated by other pathways (10, 62).

**Figure 10:**
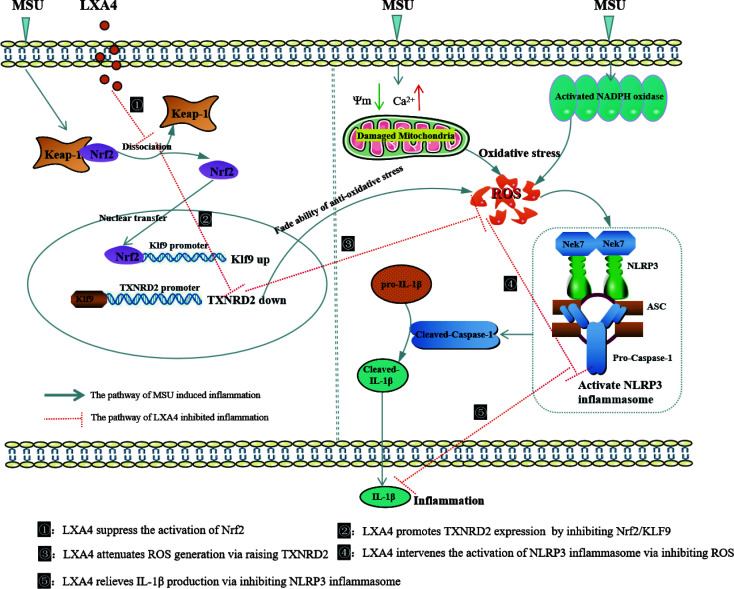
Schematic depicting a proposed model for LXA4. LXA4 alleviates MSU-crystal-induced NLRP3 inflammasome activation by suppressing Nrf2 to promote TXNRD2.

In summary, the present study demonstrates that LXA4 suppresses ROS generation, thereby attenuating NLRP3 inflammasome activation induced by the MSU crystal. Importantly, we elucidated an undescribed regulatory mechanism for LXA4 in attenuating oxidative stress and NLRP3 inflammasome activation. As illustrated in **Figure 10**, LXA4 suppresses Nrf2 activation and then decreases Klf9 expression, thereby promoting TXNRD2 expression, which is conducive to LXA4 inhibiting ROS generation and preventing subsequent NLRP3 inflammasome activation. Additionally, the results from *in vivo* animal models support the expectation that LXA4 may be an attractive new candidate for the treatment of gouty arthritis.

## Data availability statement

The original contributions presented in the study are included in the article/**Supplementary Material**. Further inquiries can be directed to the corresponding author.

## Ethics statement

This study was approved by the Ethics Committee of the Lanzhou University Second Hospital, and all patients provided informed consent.

## Author contributions

CY, YZ, XG and XZ designed the study. YZ and LX performed the experiments in this work. YZ, YC, HX, MYZ, MCZ, analyzed the data in this study. YZ wrote the manuscript. All authors contributed to the article and approved the submitted version.

## Supplementary material

Supplementary Figure 1 BMDMs and PMA differentiated THP-1 macrophages were primed with LPS (300 ng/ml) for 3 h and then pretreated with LXA4 (50-150 nM) for 1 h; subsequently, 100 μg/ml MSU crystals were added for 24 h. **(A, B)** Cell lysates were analyzed by immunoblotting to determine the NEK7 protein levels in BMDMscell and PMA differentiated THP-1 macrophages. **(C, D)** The interaction between NEK7 and NLRP3 was analyzed by immunoprecipitation using an antibody against NEK7 and then measured by immunoblotting in BMDMs cells and PMA-differentiated THP-1 macrophages.

Supplementary Figure 2 The PMA-differentiated THP-1 macrophages were transfected with TXNRD2 siRNA and negative control siRNA. **(A)** Cell lysates were analyzed by immunoblotting to determine the TXNRD2 protein levels. **(B)** RT-qPCR was performed to measure the TXNRD2 mRNA levels (n=4). Data are presented as the mean ± SEM.

Supplementary Figure 3 The PMA differentiated THP-1 macrophages were primed with LPS (300 ng/ml) for 3 h and then pretreated with LXA4 (150 nM) and AUR (1 μM) for 1 h; subsequently, 100 μg/ml MSU crystals were added for 24 h. **(A)** Supernatants were analyzed by ELISA to measure IL-1β (n=3). **(B)** Supernatants and cell lysates were analyzed by immunoblotting to determine the release of cleaved caspase-1 and matured IL-1β. Data are presented as the mean ± SEM, ^**^ *P*<0.01 compared with the vehicle group, ^#^ *P*<0.05 compared with the MSU group.

Supplementary Figure 4 The PMA differentiated THP-1 macrophages were transfected with TXNRD2 siRNA before being primed with LPS and then pretreated with LXA4 (150 nM) for 1 h; subsequently, 100 μg/ml MSU crystals were added for 24 h. The percentage of cells with ASC-speck relative to all cells was calculated (n=3). Data are presented as the mean ± SEM, ^*^ *P*<0.05 compared with the vehicle group, ^#^ *P*<0.05 compared with the MSU group.
