# Supplementary material for: Lipoxin A4 attenuates MSU-crystal-induced NLRP3 inflammasome activation through suppressing Nrf2 thereby increasing TXNRD2
Source: Front Immunol. 2022 Dec 8;13:1060441. doi: 10.3389/fimmu.2022.1060441 (PMC9772058; doi:10.3389/fimmu.2022.1060441)
Supplement: Supplementary file 1 [file DataSheet_1.doc]

**Supplementary materials**

**Antibodies**: The primary antibodies used for mouse, human and rat experiments were anti-IL-1β(WL02257)(1:1000, WanleiBio, shenyang, chinaA), anti-Caspase-1(WL03325)(1:1000, WanleiBio, shenyang, china), anti-NLRP3(WL02635)(1:1000, WanleiBio, shenyang, china), and anti-β-actin(GTX109639)(1:5000, GeneTex, CA, USA). Anti-GSDMD(39754)(1:1000, Cell Signaling Technology, Danvers, MA, USA), anti-ASC(sc-514414)(1:1000, Santa, Dallas, TX, USA), anti-Nek79sc-393539)(1:1000, Santa, Dallas, TX, USA), anti-α-Tubulin(GTX112141)(1:5000, GeneTex, CA, USA), anti-GAPDH(GTX100118)(1:5000, GeneTex, CA, USA), anti-Histone H3(WL0984a)(1:1000, WanleiBio, shenyang, china) were used to measure mouse and human protein. The primary antibodies against human antigens were anti-p22phox(sc-271968)(1:1000, Santa, Dallas, TX, USA), anti-p47phox(sc-17845)(1:1000, Santa, Dallas, TX, USA), anti-Na+-K+-ATPase(YT2971)(immunoway, suzhou, china), anti-Nrf2(sc-365949)(1:1000, Santa, Dallas, TX, USA), anti-HO-1(1:1000, WanleiBio, shenyang, china), anti-NQO1(YT3186)(immunoway, suzhou, china), anti-SOD2(WL02506)(1:1000, WanleiBio, shenyang, china), anti-GPX(WL02497a)(1:1000, WanleiBio, shenyang, china), anti-Klf9(sc-376422)(1:1000, Santa, Dallas, TX, USA), anti-TXNRD2(YT4752)(immunoway, suzhou, china). The secondary HRP-conjugated antibodies were anti-mouse IgG(BM2020)(1:5000, Boster Bio, Wuhan, china) and anti-rabbit IgG(BA1041)(1:5000, Boster Bio, Wuhan, china).
