# Supplementary material for: Lipoxin A4 attenuates MSU-crystal-induced NLRP3 inflammasome activation through suppressing Nrf2 thereby increasing TXNRD2
Source: Front Immunol. 2022 Dec 8;13:1060441. doi: 10.3389/fimmu.2022.1060441 (PMC9772058; doi:10.3389/fimmu.2022.1060441)
Supplement: Supplementary file 2 [file Table_1.doc]

**Supplementary Table 1**

**The sequences of this study.**

| gene | sequence | |
| --- | --- | --- |
| Klf9 | Forward sequence (5′–3′) | CTGGTTGCTGGGACTGTAGC |
|  | Forward sequence (5′–3′) | GTTTTCCAGCTCCCAAACAG |
| TXNRD2 | Forward sequence (5′–3′) | CCTTTGACACCGTCCTGTG |
|  | Forward sequence (5′–3′) | GAAGGCGAGGATGATTGC |
| HO-1 | Forward sequence (5′–3′) | CCCACCAAGTTCAAACAGCTC |
|  | Forward sequence (5′–3′) | AGGAAGGCGGTCTTAGCCTC |
| SOD | Forward sequence (5′–3′) | CACTGCAAGGAACAACAGGC |
|  | Forward sequence (5′–3′) | ACCAGGCTTGATGCACATCTT |
| GPx | Forward sequence (5′–3′) | TTATTAACGATGTCCAACCCGTC |
|  | Forward sequence (5′–3′) | CCAGAGCTATGCCAACAAAATCT |
| β-actin | Forward sequence (5′–3′) | AGCCTCGCCTTTGCCGA |
|  | Forward sequence (5′–3′) | CTGGTGCCTGGGGCG |
| TXNRD2 siRNA | 5′–3′ | CTTGGAATATGGAATCACA |
