# Supplementary figures and images for: Lipoxin A4 attenuates MSU-crystal-induced NLRP3 inflammasome activation through suppressing Nrf2 thereby increasing TXNRD2
Source: Front Immunol. 2022 Dec 8;13:1060441. doi: 10.3389/fimmu.2022.1060441 (PMC9772058; doi:10.3389/fimmu.2022.1060441)

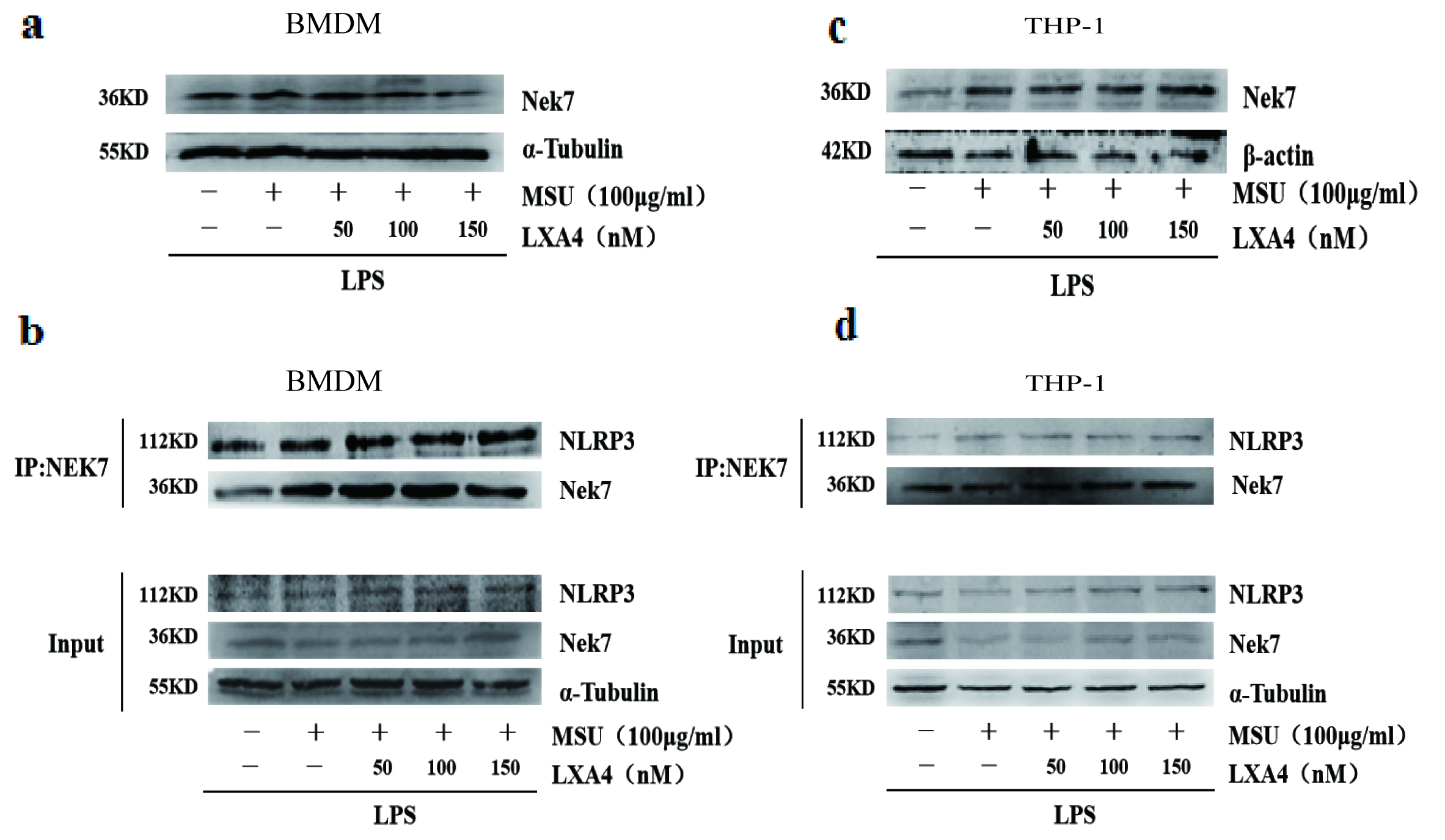

Supplement: Supplementary Figure 1 — BMDMs and PMA differentiated THP-1 macrophages were primed with LPS (300 ng/ml) for 3 h and then pretreated with LXA4 (50-150 nM) for 1 h; subsequently, 100 μg/ml MSU crystals were added for 24 h. (A, B) Cell lysates were analyzed by immunoblotting to determine the NEK7 protein levels in BMDMscell and PMA differentiated THP-1 macrophages. (C, D) The interaction between NEK7 and NLRP3 was analyzed by immunoprecipitation using an antibody against NEK7 and then measured by immunoblotting in BMDMs cells and PMA-differentiated THP-1 macrophages. [file Image_1.tif]

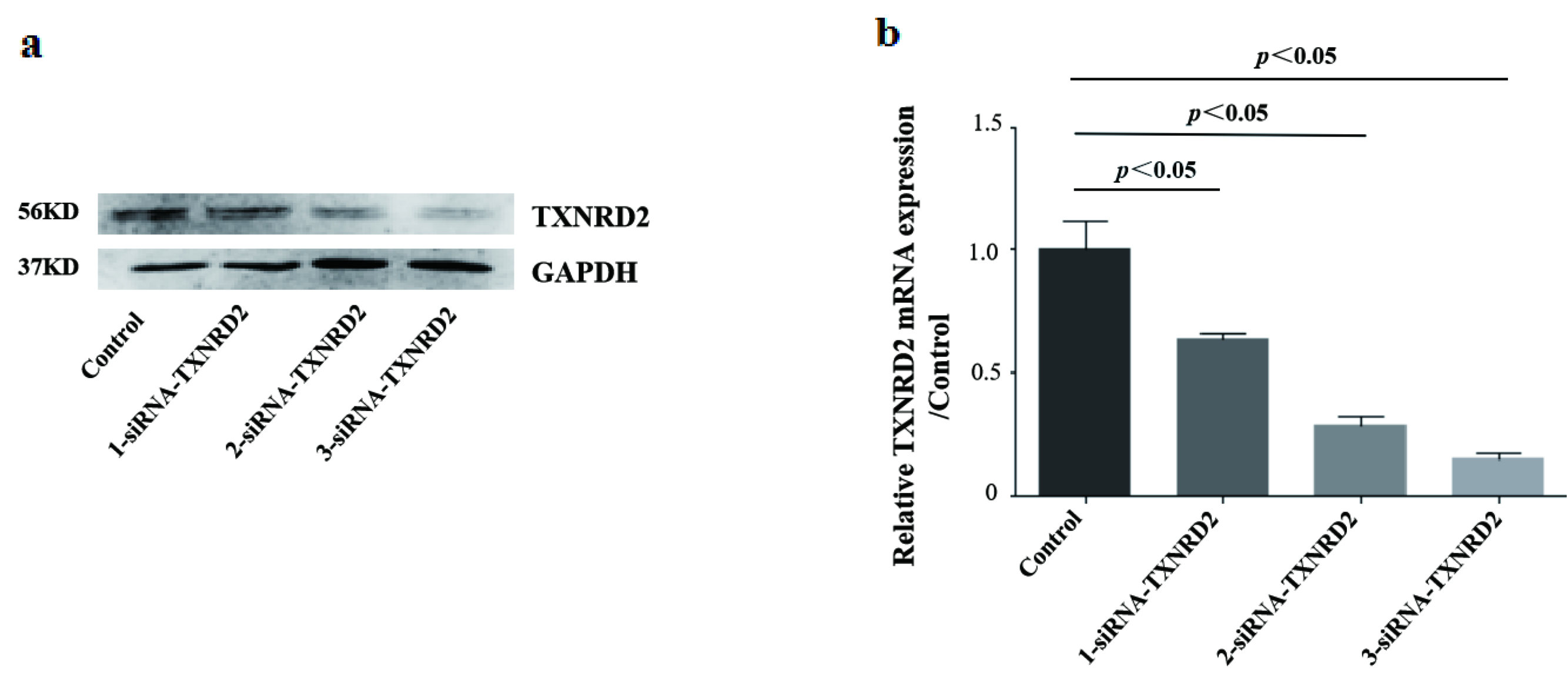

Supplement: Supplementary Figure 2 — The PMA-differentiated THP-1 macrophages were transfected with TXNRD2 siRNA and negative control siRNA. (A) Cell lysates were analyzed by immunoblotting to determine the TXNRD2 protein levels. (B) RT-qPCR was performed to measure the TXNRD2 mRNA levels (n=4). Data are presented as the mean ± SEM. [file Image_2.tif]

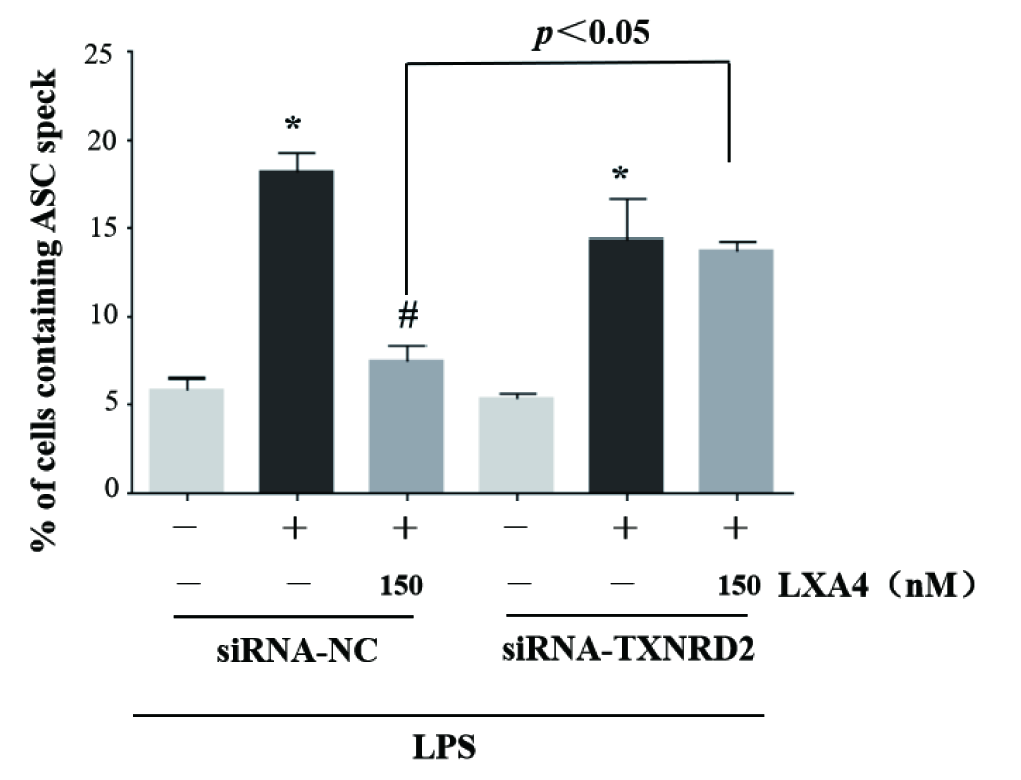

Supplement: Supplementary Figure 3 — The PMA differentiated THP-1 macrophages were primed with LPS (300 ng/ml) for 3 h and then pretreated with LXA4 (150 nM) and AUR (1 μM) for 1 h; subsequently, 100 μg/ml MSU crystals were added for 24 h. (A) Supernatants were analyzed by ELISA to measure IL-1β (n=3). (B) Supernatants and cell lysates were analyzed by immunoblotting to determine the release of cleaved caspase-1 and matured IL-1β. Data are presented as the mean ± SEM, ** P<0.01 compared with the vehicle group, # P<0.05 compared with the MSU group. [file Image_3.tif]

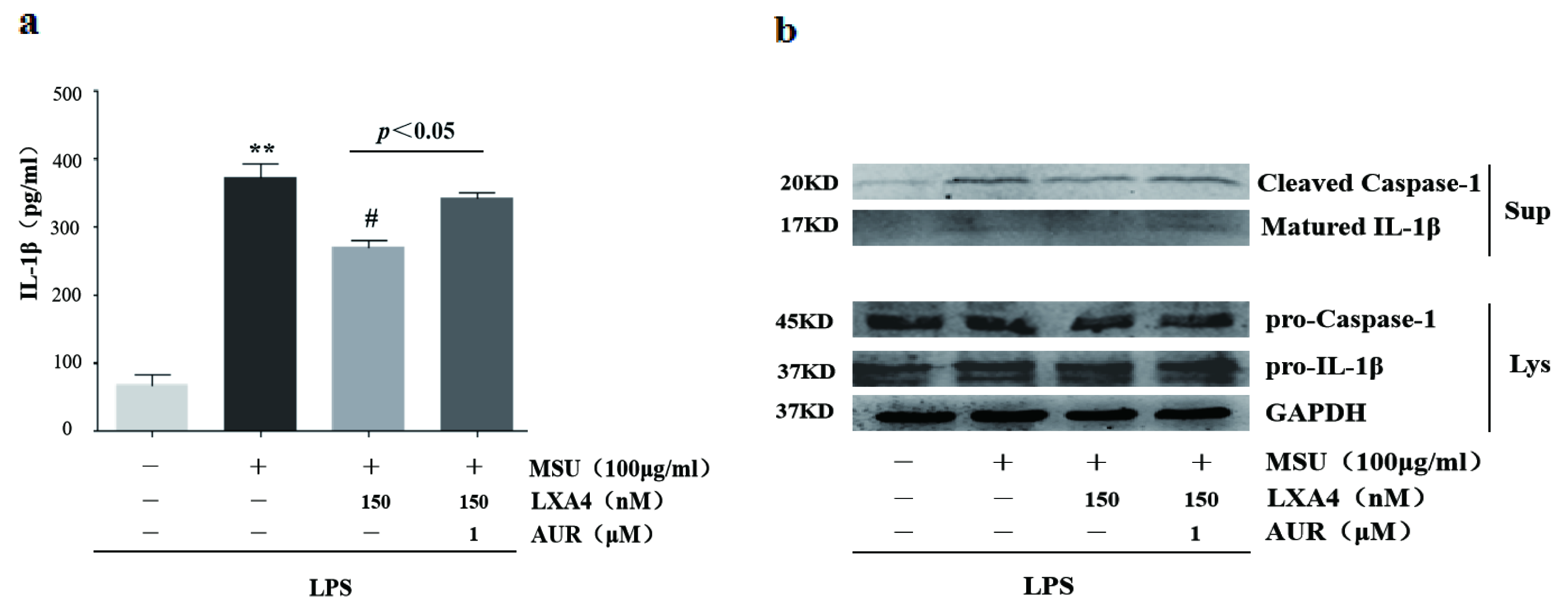

Supplement: Supplementary Figure 4 — The PMA differentiated THP-1 macrophages were transfected with TXNRD2 siRNA before being primed with LPS and then pretreated with LXA4 (150 nM) for 1 h; subsequently, 100 μg/ml MSU crystals were added for 24 h. The percentage of cells with ASC-speck relative to all cells was calculated (n=3). Data are presented as the mean ± SEM, * P<0.05 compared with the vehicle group, # P<0.05 compared with the MSU group. [file Image_4.tif]
